# Raising the Sugar Content – Orchid Bees Overcome the Constraints of Suction Feeding through Manipulation of Nectar and Pollen Provisions

**DOI:** 10.1371/journal.pone.0113823

**Published:** 2014-11-25

**Authors:** Tamara Pokorny, Klaus Lunau, Thomas Eltz

**Affiliations:** 1 Department of Animal Ecology, Evolution and Biodiversity, University of Bochum, Bochum, Germany; 2 Institute of Sensory Ecology, University of Düsseldorf, Düsseldorf, Germany; San Diego, United States of America

## Abstract

Unlike most other bees, the long-tongued orchid bees ingest nectar using suction feeding. Although long tongues allow exploitation of flowers with deep spurs, the energy intake rate is optimal at 10–20% lower nectar sugar concentrations compared to that of lapping bees. This constraint might be compensated by a higher digestive throughput. Additionally, orchid bees might evaporate water from regurgitated droplets of crop contents. We found male *Euglossa championi* (n = 10) and *Euglossa dodsoni* (n = 12) to regularly regurgitate droplets of crop content to the base of their proboscis, generating a fluid film between the proximal parts of the galeae, glossa and labial palps. Rhythmic movements of the proboscis may help to increase convection. There was a significant change in sugar concentration between the initially imbibed solution and the resulting crop content (*P*<0.05) and the time individual bees had engaged in this liquid exposure behavior was positively correlated with the resulting crop sugar concentration. Female *Euglossa townsendi* and *Euglossa viridissima* showed the same behavior. Additionally, they manipulated their nectar-enriched pollen provisions for extensive periods of time before deposition in brood cells. The deposited pollen loads (n = 14) showed a significantly higher sugar concentration than the sugar-water available to the bees (*P*<0.001). Thus, both male and female euglossines show behaviors that promote evaporative water loss from nectar. We suggest that the behaviors have evolved in concert with suction feeding on dilute nectar from deep floral tubes.

## Introduction

Carbohydrates in form of nectar sugars constitute the main energy source for adult bees [1], [2]. Most bees ingest nectar by lapping, with the maximal energy intake rate being achieved for sugar concentrations of around 50–60% [3], [4]. Lower and higher concentrations can be imbibed as well, and it has been shown in honey bees that nectar throughput through the proventriculus is adjusted to the nectar sugar concentration and the metabolic rate, with lower concentrations receiving a relatively higher throughput and vice versa [5]. Though this mechanism ensures a consistent energy supply independent of the concentrations of imbibed solutions, honey bees and other bees usually prefer the higher concentrated nectar if given a choice (honey bees [6]; euglossine bees [7]; bumble bees [8]). A high sugar to water ratio of crop content means that the carried weight has more energetic value, which would allow the bees to remain active and airborne for longer periods of time without having to refuel at nectar sources. This could be especially beneficial for activities that require high energy expenditure (flying/hovering) over reasonably long periods of time, when either access to flowers is not possible, or the time is required for other behaviors (discussed in e.g. [9]) This can be envisioned to apply in the context of mate-finding, e.g. during mating flights in carpenter bees [10] or bumble bees [11], where males stay airborne for long periods of time, or when defending a territory (see [12] Discussion). Other situations in which bees should benefit from energetically rich crop contents are fast long range movements and when female bees forage for pollen at plants that do not provide nectar (see [13]).

Some bees have been reported to achieve energetically more favorable sugar concentrations of crop content by evaporating water from regurgitated droplets of nectar. They extend the proboscis with a droplet of regurgitated nectar visible either around the mandibles, the outstretched galeae or at the tip of the proboscis with the glossa protruding into it. Often either the glossa or the proboscis is moved, and the droplet is re-ingested and regurgitated repeatedly (nectar dehydration or ‘bubbling’; honey bees [14]; carpenter bees [9], [15], [16]; bumble bees [11]; stingless bees [17]; allodapine bees [18]; colletid bees [19]; halictine bees [20]).

Euglossine bees (Hymenoptera, Apidae, Euglossini), important Neotropical pollinators of a variety of plants [21], are characterized by their very long proboscides [21], which allow them to exploit flowers with long tubes in addition to other flower types [22], [23]. In contrast to other bees, however, the orchid bees employ suction feeding for nectar ingestion [24]. As viscosity rises exponentially with the sugar concentration [25], [26], the suction feeding style is constrained by nectar concentration. This is visible in the fact that the optimal energy intake rate is achieved for approximately 10–20% lower sugar concentrations (30–40%) compared to the lapping style ([24]; reviewed by [27]). Accordingly, such more dilute nectars are often found in plants with deeply corollated flowers visited by orchid bees ([28]; and see [29], [30] on crop samples of orchid bees). Orchid bees are nevertheless strong flyers, easily capable of rapid flight and covering long distances (23 km in one day [31]; 45 km within two days and distances of up to 95 km [32]). Males can be observed collecting volatile components for pheromone analogs (see [21], [33], [34], [35]), showing territorial display behavior and engaging in territorial contests with other males continuously for long durations of time (contests can last up to over an hour [36]; D. W. Roubik, pers. comm.; T. Pokorny, pers. obs.). The importance of sugar concentration for maintaining high metabolic activity raises the question on how orchid bees manage to overcome the constraint of suction feeding in order to cover their energy expenditure on predominantly dilute nectars. A higher throughput of nectar seems improbable to be the only factor accountable for such demanding flight accomplishments, especially in the light of the long timespans without visits to nectar sources (>1 h, see [36]: contest duration; [37]: odor collection). Nectar dehydration in the same manner as employed by other bees is rather unlikely. First, the euglossine proboscis is too long for such a procedure, as it reaches at least sternum I when held under the body and can in some cases even reach beyond the tip of the metasoma [21]. Stretching the proboscis out with the droplet at the tip or around the whole proboscis, as described for other bees, therefore seems improbable. Second, reuptake of highly concentrated nectar droplets from the tip of the proboscis would likely be constrained by the increased viscosity of the dehydrated nectar (see [24]). Nevertheless, nectar water evaporation might be achieved by comparable means. In cage settings, male and female *Euglossa* spp. can be seen showing a conspicuous behavior reminiscent of that of nectar dehydration in other bees, which will henceforth be called ‘tongue flicking’. Tongue flicking in orchid bees differs from evaporation behaviors described for other bees in that the proboscis stays located beneath the bees’ body, and the regurgitated liquid is stretched out as a thin film between the different proximal parts of the proboscis. In this study we assessed whether the tongue flicking behavior serves to evaporate water from crop content for males of two sympatric species of *Euglossa* (*E. championi* and *E. dodsoni*) that differ in size (13 mm/10 mm) and proboscis length (extended 16 mm/18.7 mm, see [21]).

Sugar concentration does not only have an impact on the adults’ energy budget, but plays an important role in brood provisions as well (reviewed in [38]). All solitary and most eusocial bees (excepting honey bees) mass provision their brood cells, sealing them after the egg has been laid. Provisions commonly consist of pollen with more or less added nectar [15], [39], [40], [41]. The added nectar constitutes up to 99% of the sugar content of provisions [41], and higher sugar contents in larval provisions have been shown to positively influence the resulting larval weight [42], [43]. Higher larval weight was discussed as potential factor increasing adult mass and fecundity [43]. Based on this, we investigated whether female *Euglossa* orchid bees have behavioral adaptations relating to the manipulation of sugar concentrations of brood provisions. Here, we focused on *E. viridissima* and *E. townsendi*, two species that readily accept trap nests and thus can be obtained for observation.

## Materials and Methods

**Figure 1:**
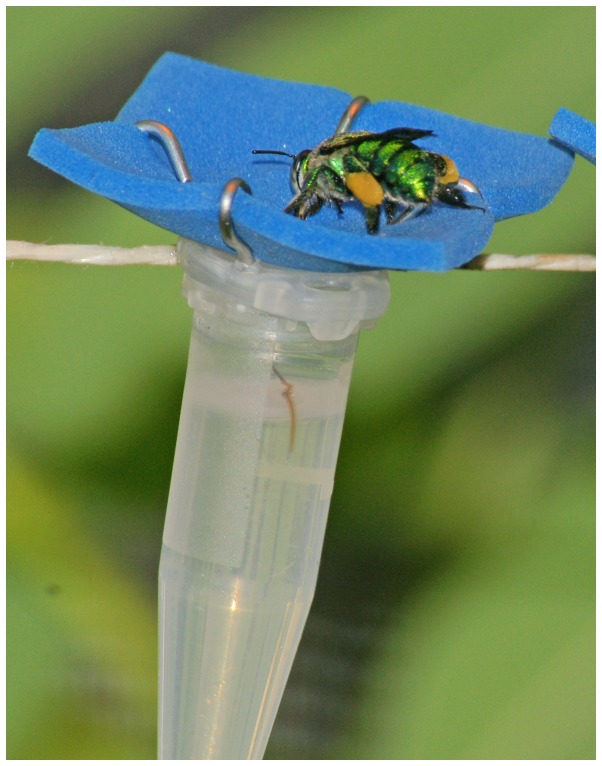
Artificial flowers. Female *Euglossa viridissima* drinking from an artificial flower (constructed from a 1.5 ml Eppendorf tube and foam rubber) containing sugar-water solution. The tip of the glossa can be seen just above the 1 ml mark of the Eppendorf tube.

Experiments on nectar dehydration were conducted during May 2013 at the Tropical Station La Gamba, Puntarenas, Costa Rica. We used male bees as they can easily be captured in sufficient numbers and cannot sting, facilitating handling and thus minimizing handling time (Field work permission granted by the Ministerio del Ambiente y Energía, Sistema Nacional de Areas de Conservación, Costa Rica). Male *E. championi* and *E. dodsoni*, like many euglossine males, can be easily attracted to scent baits where they were captured using hand nets and transferred into Eppendorf vials with breathing holes. After return to the field station, they were individually marked with Opalithplättchen (Graze, Germany) of different colors and shapes and released together into a mesh cage (45×45×45 cm) outfitted with perching opportunities (branches with leaves) and feeders in the shape of artificial flowers (see Fig.1), the latter of which were removed each evening to avoid fermentation. They were trained to take sugar-water (sucrose dissolved in water, 30%w/w) from the artificial flowers over the course of three to five days. The experiments began when all remaining bees (*E. championi*: n = 10; *E. dodsoni*: n = 12) were sufficiently familiar with the cage situation and could be expected to show normal behavior (i.e. not constantly flying against the mesh walls searching for a way out and not spending hours trying to pry the Opalithplättchen off the mesosoma). At 0510 hours, before first morning activity, artificial flowers were placed in the cage. All flowers contained the same concentration of sugar-water solution. Observation began with first drinking activity between 0525 and 0535 hours and lasted five hours. The duration (in minutes) of time spent drinking and that spent tongue flicking was noted for each individual. After the observation period each bee was captured, and a sample of its crop content was obtained by gently squeezing the metasoma and taking up the regurgitated droplet using glass microcapillaries (2 µl). Bees were immediately released back into the cage where they were allowed to feed freely for the rest of the day. Samples of crop content were diluted by four with distilled water in order to obtain a sufficient amount of liquid to be measured with a handheld refractometer (Müller, Germany) corrected for temperature. Resulting values were corrected for the dilution. Six different sugar concentrations lying within the possible feeding range of orchid bees were offered on consecutive days in the following order: 40.8%–30.0%–34.6%–45.2%–25.0% and 50.0% sugar (order determined randomly with the restriction that the lowest and highest sugar solution would be tested last). Each sugar solution was freshly prepared on the respective morning, and measured for its sugar concentration using the handheld refractometer prior to filling the artificial flowers with the mixture. Concentrations deviated from the targeted 5%-steps (three cases, 40.8 instead of 40%, 34.6 instead of 35% and 45.2 instead of 45%) when adjustment of sugar concentration was not accomplished in time before the feeders had to be exposed. All ten *E. championi* and twelve *E. dodsoni* were tested on each of the different concentrations. Additionally, samples from the artificial flowers were taken in the evenings to monitor evaporative water loss from the provided sugar-water.

Female *E. viridissima* and *E. townsendi* reared from trap nests were observed during the autumn months of 2012 in a large flight cage (8.8×3.3×2.8 m) kept under greenhouse conditions in Düsseldorf, Germany. The bees could feed ad libitum from artificial flowers filled with sugar solution (30–36%) which was exchanged daily. *Solanum lycopersicum*, *Senna alata* and *Senna bicapsularis* were offered as pollen sources, as preliminary tests had shown their suitability as pollen sources for these bees. Additionally, hardwood nest boxes (5.5×10.0×3.3 cm) and stingless bee cerumen were provided for nest construction. Pollen manipulation outside of the nest boxes was documented on several occasions. When possible, samples of freshly deposited pollen loads were obtained for analysis of sugar concentration. Samples were weighed, diluted with demineralized water and mixed using a vortex (Reax Top, Heidolph, Germany) in order to solubilize the contained sugar. When all of the pollen was suspended in the water, the samples were centrifuged (Micro 20, Hettich, Switzerland, 13000 rpm for 10 minutes), and the sugar content of the supernatant was measured using a handheld refractometer (Krüss Optronic, Germany). Resulting values were corrected for the dilution.

### Statistical analyses

We tested whether sugar concentration differed between the offered solution and crop samples after tongue flicking as well as between offered solution and the liquid part of brood provisions after pollen manipulation using Wilcoxon signed rank tests for paired data. Welch two sample t-tests served to compare the average differences between initial and resulting sugar concentration with or without tongue flicking, and correlation of tongue flicking time with resulting sugar concentration of crop content as well as correlation of initial concentration with tongue flicking time were analyzed with Spearman rank correlations. All tests were performed in R v.3.0.2 [44].

## Results

### Description of behaviors

**Figure 2:**
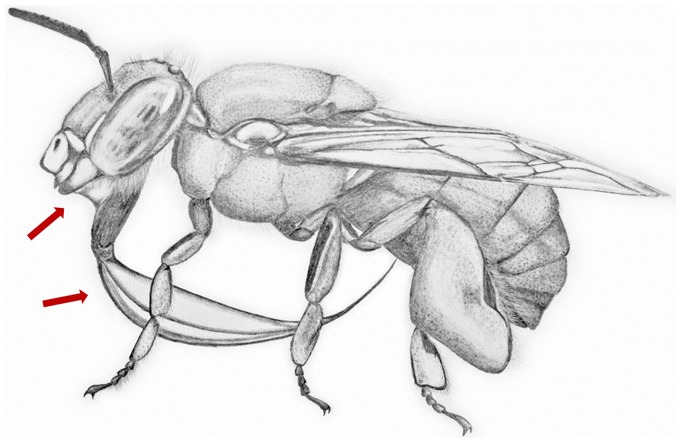
Areas of fluid exposure during tongue flicking behavior. Schematic drawing of the posture during tongue flicking behavior. The proximal part of the proboscis is split into galeae, glossa and labial palps, with a fluid film stretched in between and a droplet of regurgitated nectar positioned between the mandibles and the base of the proboscis, indicated by the arrows.

Tongue flicking: After feeding from the artificial flowers, the bees commonly withdrew to secluded places in the cage, where they perched nearly motionless with the proximal part of the proboscis split into galeae, glossa and labial palps, drawing a fluid film between them (Fig.2). Additionally, a large drop of liquid was often visible around the mandibles and the upper part of the proboscis, which was moved back and forth rhythmically (see Video S1).

**Figure 3:**
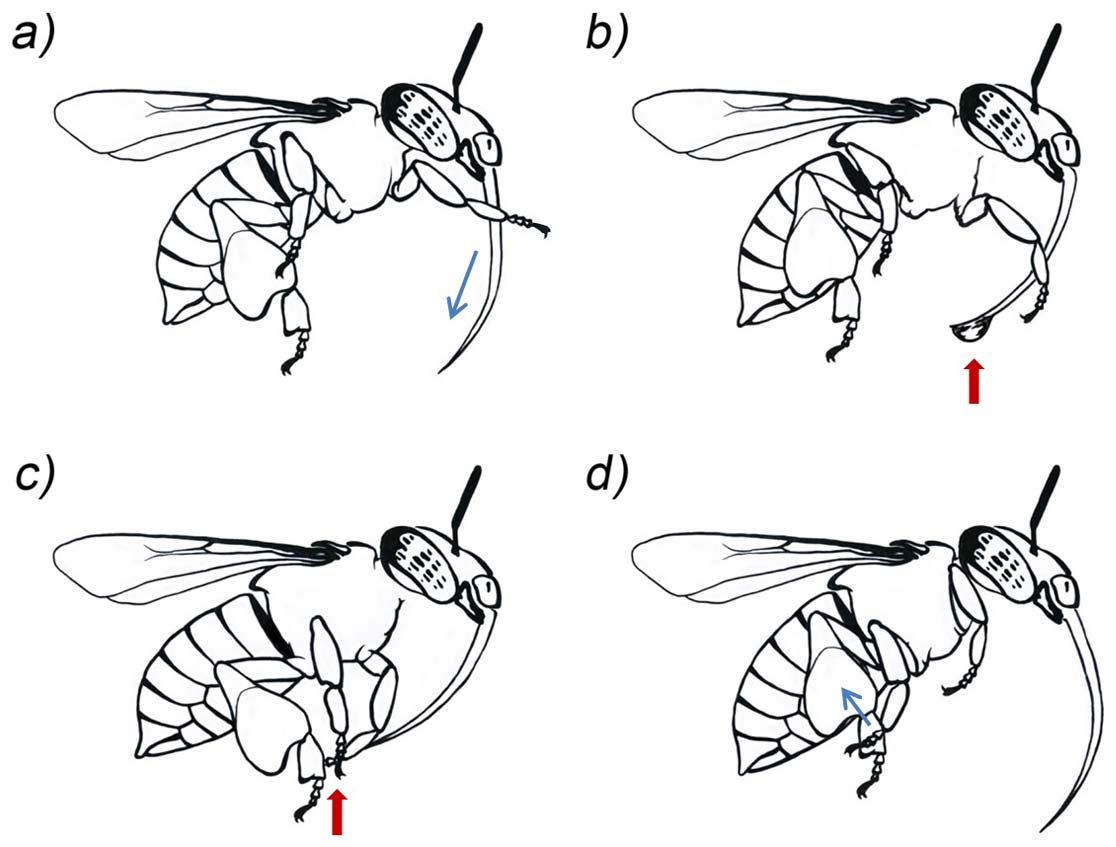
Additional moistening of pollen provisions. Schematic rendering of four video stills (from Video S2) depicting the addition of a regurgitated droplet of sugar-water to the pollen provision carried on the corbiculae while hovering. a) Placement of both front basitarsi at the base of the outstretched proboscis. Basitarsi are thereafter pulled downward to the tip of the proboscis in one swiping movement. b) Appearance of a droplet at the tip of the proboscis. c) Uptake of the droplet with the front tarsi and subsequent relocation to the middle tarsi. d) Transfer of the droplet from the middle tarsi to the pollen provisions by moving them upward from the hind basitarsi.

Application of sugar-water to the pollen load: In the course of pollen collection and manipulation, female bees added regurgitated droplets to their corbiculae by extending the proboscis and positioning both front basitarsi on either side, moving them down along the proboscis. When the front legs were about a third of the way down, a droplet would start to appear at the tip of it. The droplet would eventually be swiped off the tip of the proboscis with the front tarsi. Subsequently, this droplet was taken up from there by the middle tarsi, which were positioned on the hind basitarsi and then moved upward onto the corbiculae, thus eventually transferring the droplet to the pollen load (see Fig.3). Additional patting movements of the middle legs on the pollen load could be observed after wiping the front tarsi off a second time (see Video S2).

**Figure 4:**
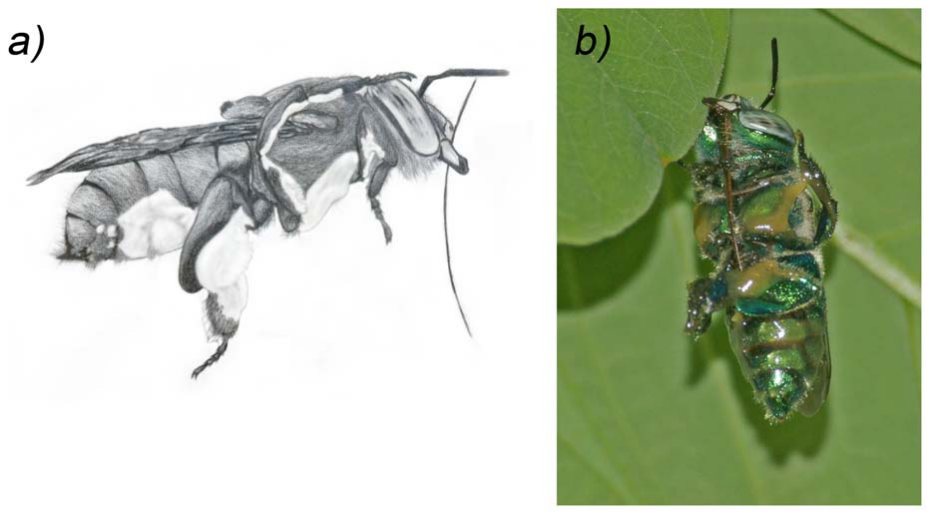
Manipulation of pollen provisions. Posture of a female *E. viridissima* during manipulation of nectar enriched pollen provisions, kneading. a) Schematic drawing of lateral view, with the nectar-pollen mixture rendered in white. b) Ventral view with the nectar-pollen mixture spread across the ventral meso- and metasoma as well as the legs.

Kneading: After having filled their corbiculae, the females withdrew to a secluded perch, mostly positioned near the top of the cage, where they attached themselves beneath or on the side of a leaf by their mandibles. Due to this, observation never included the first few seconds of the further pollen manipulation process. For pollen manipulation, kneading, the females took up a characteristic posture (Fig.4) with the moistened pollen spread across the ventral side of the metasoma, the corbiculae and the ventral side of the middle legs. Sometimes small amounts of nectar-pollen mixture would also be present on the front legs. The middle legs were held up at a right angle to the body with the tarsi facing forward for most of the time. The hind legs made up-and-down pumping motions, moving the basitarsi together and away from each other in elliptical paths. In regular intervals the middle legs were brought down onto the pollen mass, patting and ‘kneading’ it, and then lifted up into position again. All the while the front legs were held motionless beside the head (see Video S3). Occasionally more droplets of liquid were added to the mass. In the end, the pollen was groomed off the bees’ body and transferred back to the corbiculae, forming smooth, shiny pollen loads. The whole process from end of pollen collection until entering the nest with the manipulated pollen loads took on average 24 minutes (n =  ten observations, SD = 9 minutes).

### Tongue flicking and nectar dehydration

**Figure 5:**
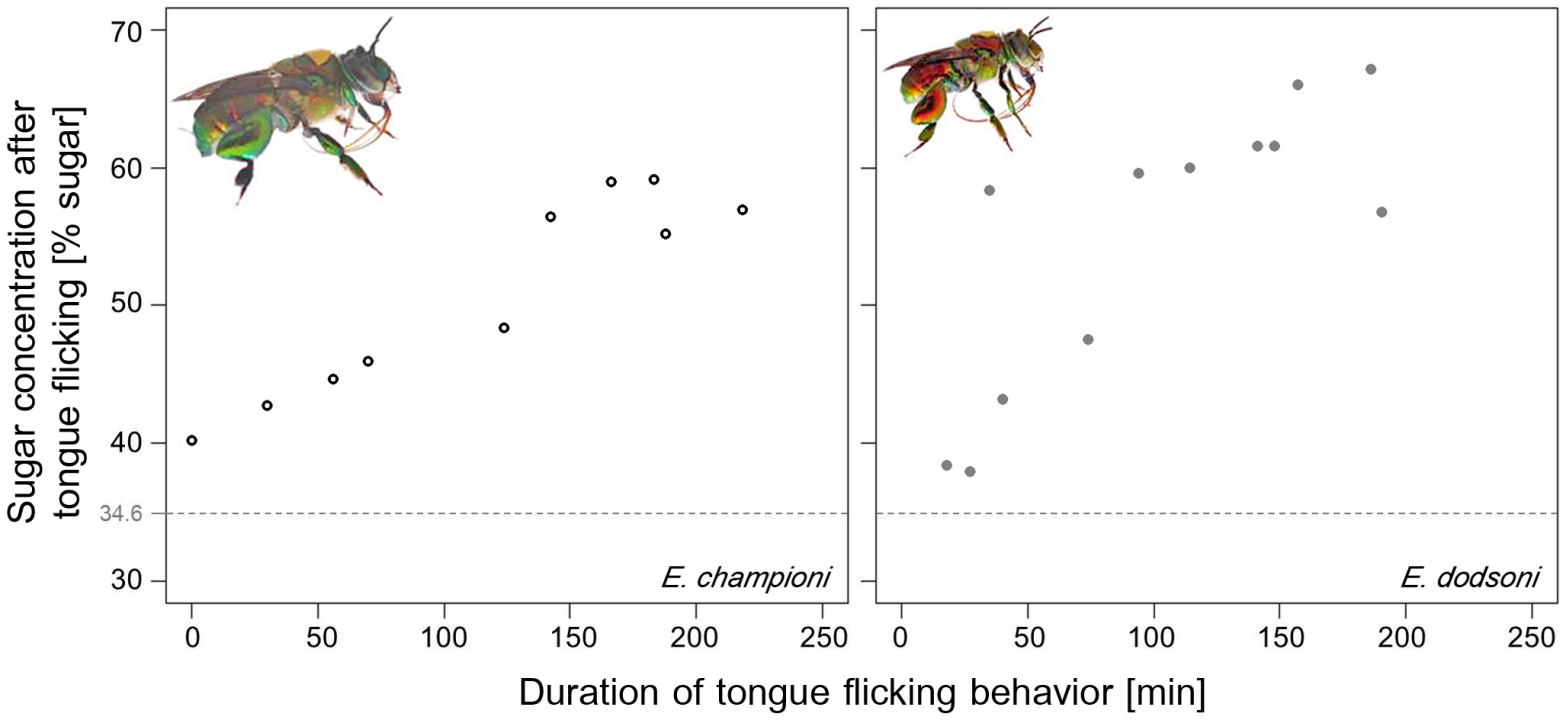
Positive correlation between tongue flicking behavior and resulting sugar concentration of crop contents. Exemplary presentation of the results for an initial sugar concentration of 34.6%. Left graph: *E. championi*: Spearman rank correlation, n = 10, *P*<0.01, rho = 0.87; right graph: *E. dodsoni*: Spearman rank correlation, n = 12, *P*<0.01, rho = 0.76. Each circle or dot represents the values for a different individual male.

Male (Costa Rica and Germany Greenhouse) as well as female (Germany Greenhouse) *Euglossa* spp. showed the tongue flicking behavior. Each of the male bees in Costa Rica did so on at least two of the six days they were observed. There were no obvious differences between the two species regarding results on nectar evaporation despite the differences in body size and relative proboscis length. They spent between 0 and 234/245 minutes (*E. championi* and *E. dodsoni*, respectively, see Table 1) of the 300 minute observation period showing tongue flicking behavior. There was no correlation between the offered sugar concentration and the duration of the tongue flicking behavior (Spearman rank correlation, *E. championi*: *P* = 0.3068, rho  = 0.1341; *E. dodsoni*: *P* = 0.9007, rho  = −0.0149, Table 1). Resulting crop sugar concentrations were always higher than those that had been offered in the artificial flowers (one exception, see Table 1), with a significant change in sugar concentration after tongue flicking for all tested concentrations and both species (Wilcoxon signed rank test for paired data, *P*<0.05, see Table 2). The sugar concentration of crop contents reached values as high as 72.6% for *E. championi* and 75.6% for *E. dodsoni*, up to 34.8% higher than the originally imbibed concentration. Average values for resulting sugar concentrations of crop contents were lower due to a general variability in duration and efficiency of tongue flicking behavior, and some individuals did not show tongue flicking behavior at all on some of the days (see Table 1), but remained generally inactive, spending most of the observation time hanging by their mandibles from leaves or the mesh walls in a torpor-like state. In cases in which individuals had not engaged in tongue flicking behavior, crop content concentration was on average 6.8% (*E. championi*, n = 5 cases, SD: 2.0%) and 4.8% (*E. dodsoni*, n = 17 cases, SD: 4.5%) higher than the respective feeder sugar concentration. This was however significantly lower than the average concentration difference of crop content to offered sugar-water in all cases in which tongue flicking occurred (14.9%, SD: 6.8%, and 19.5%, SD: 9.5%; Welch two sample t-test: *E. championi*, t = 6.2966, df = 15, *P*<0.001; *E. dodsoni*, t = 8.7303, df = 58, *P*<0.001). Additionally, duration of tongue flicking behavior and resulting concentration were positively correlated (Fig.5, Table 2) in all but one case (30% initial concentration for *E. championi*, Spearman rank correlation, *P* = 0.1909, see Table 2). Evaporative water loss from the artificial feeders during the respective days led to values maximally 1% higher than the sugar concentration measured in the morning (Table 2), and could therefore not have been responsible for the higher concentrations found in the crop contents.

**Table 1:** Observed duration of tongue flicking (min, first column for each initial concentration) and resulting sugar concentration (%, second column for each initial concentration) of crop content samples of the ten *E. championi* and twelve *E. dodsoni* individuals for each of the six offered initial concentrations.

% Sugar Bee #		25% *(25.5%)*		30% *(30.4%)*		34.6% *(35.1%)*		40.8% *(41.8%)*		45.2% *(45.2%)*		50% *(50.8%)*	
Bee #		t(min)	sugar%	t(min)	sugar%	t(min)	sugar%	t(min)	sugar%	t(min)	sugar%	t(min)	sugar%
***E. championi***	**I**	86	32.4	60	43.6	124	48.4	54	54.2	45	54.8	5	52.4
	**II**	1	32.6	45	38.4	30	42.8	37	56.4	62	58.0	58	58.8
	**III**	89	35.8	52	32.8	70	46.0	0	50.0	21	55.2	63	59.4
	**IV**	141	51.4	234	58.8	166	59.0	36	53.4	171	69.6	225	68.2
	**V**	116	58.6	49	44.6	183	59.2	102	65.6	99	66.0	122	72.6
	**VI**	172	41.6	34	49.4	218	57.0	0	49.2	129	62.2	200	69.8
	**VII**	154	43.4	58	45.6	188	55.2	33	56.2	183	61.2	185	62.6
	**VIII**	8	34.4	116	44.4	142	56.4	116	62.6	167	56.8	179	62.2
	**IX**	0	29.2	30	41.4	0	40.2	39	57.2	9	51.8	4	53.4
	**X**	90	34.0	0	36.6	56	44.6	64	60.0	117	56.4	103	57.0
***E. dodsoni***	**I**	62	36.8	143	58.4	141	61.6	195	72.4	113	65.6	154	67.2
	**II**	43	48.4	67	71.2	35	58.4	22	61.6	87	64.0	51	58.0
	**III**	0	27.6	0	38.4	18	38.4	0	48.0	109	50.0	0	56.8
	**IV**	11	44.8	90	53.6	157	66.0	45	73.6	64	69.6	0	49.6
	**V**	204	50.4	2	36.0	114	60.0	73	75.6	110	68.4	51	58.0
	**VI**	104	31.6	0	32.0	74	47.6	0	48.8	109	59.2	0	51.6
	**VII**	139	50.8	104	46.4	94	59.6	59	55.2	97	59.6	245	67.2
	**VIII**	34	30.0	154	53.6	190	56.8	176	65.8	110	62.4	0	51.6
	**IX**	0	33.6	0	40.8	27	38.0	33	48.0	0	46.0	0	44.8
	**X**	24	27.6	0	36.0	40	43.2	0	49.2	56	54.4	0	53.6
	**XI**	158	47.2	0	41.6	148	61.6	30	57.6	127	66.0	48	58.0
	**XII**	189	55.6	102	56.0	186	67.2	157	75.2	196	75.6	243	68.8

Initial concentrations and feeder concentration at the end of the day (in italic behind the initial values) are shown in the uppermost row

**Table 2:** Results of statistical tests on effects of tongue flicking behavior.

sugar solution	*E. championi* (n = 10)	*E. dodsoni* (n = 12)	*E. championi* (n = 10)	*E. dodsoni* (n = 12)
**25.0%**	*P* = 0.00195	*P* = 0.00252	*P* = 0.00984, rho = 0.79394	*P* = 0.00726, rho = 0.72807
**30.0%**	*P* = 0.00195	*P* = 0.00251	*P* = 0.1909, n.s. rho = 0.45455	*P* = 0.00872, rho = 0.71675
**34.8%**	*P* = 0.00195	*P* = 0.00252	*P* = 0.00268, rho = 0.86667	*P* = 0.00412, rho = 0.76007
**40.8%**	*P* = 0.00195	*P* = 0.00252	*P* = 0.00061, rho = 0.88754	*P* = 0.00931, rho = 0.71253
**45.2%**	*P* = 0.00195	*P* = 0.00049	*P* = 0.02419, rho = 0.72121	*P* = 0.04368, rho = 0.58948
**50.0%**	*P* = 0.00195	*P* = 0.01061	*P* = 0.00681, rho = 0.81818	*P* = 0.00002, rho = 0.91845

Wilcoxon signed rank test for paired data was used to test whether changes in sugar concentration occurred between offered sugar concentration and concentration of crop content after tongue flicking (columns 2, 3). Changes were significant for all tested concentrations and both species. Correlation between tongue flicking time and resulting sugar concentration was tested using Spearman rank correlation (columns 4, 5), and was significant in all cases except 30% initial concentration for *E. championi*

### Pollen manipulation

Female *E. viridissima* and *E. townsendi* that accepted the provided nest boxes would set out to build cells with the offered cerumen, after which they started foraging for pollen. Pollen collection by buzzing usually was one of the first female activities in the morning (around 0600 hours, while most male bees were still inactive), sometimes preceded by a short visit to the artificial flowers. All offered pollen plants were visited, with occasional nectaring trips to the artificial flowers during pollen foraging trips. Sugar-water was regularly applied to the pollen load on the corbiculae. We could obtain freshly deposited pollen loads from four female *E. viridissima* (from one, three, three and four brood cells for each of the respective females) and two *E. townsendi* (from one and two cells, see Table 3). Samples of different brood cells from the same individuals were treated as independent samples since the variation of sugar concentration within the nest of one female was as high or higher as that found in samples from different females. Sugar concentration of the freshly deposited pollen loads (n = 14) was significantly higher than that provided in the artificial flowers (Wilcoxon signed rank test, *P*<0.001, see Table 3. Measurements of sugar concentration were taken later in the day than collection of pollen samples in order to take potential evaporation from the artificial flowers into account). Sugar concentration of pollen loads was on average 69.59% (SD: 2.98%).

**Table 3:** Differences in sugar concentration between offered sugar-water and liquid content of freshly deposited brood provisions.

Species, individual	% Sugar solution in feeders	% Sugar in liquid part of brood provision
***E. viridissima*** **, female I**	34.0	68.2
	34.0	75.3
	34.0	69.0
	34.0	67.5
***E. viridissima*** **, female II**	34.0	75.1
	34.0	67.3
	34.0	69.2
***E. viridissima*** **, female III**	34.0	73.3
	34.0	68.7
	37.8	66.3
***E. viridissima*** **, female IV**	34.0	69.9
***E. townsendi*** **, female I**	37.8	70.0
	30.0	68.5
***E. townsendi*** **, female II**	35.2	65.9

Measurements of feeder sugar solution were taken after those of brood provisions to account for evaporation. Freshly deposited brood provisions were analyzed for sugar content once per brood cell. For most females, the provision of more than one brood cell could be analyzed.

## Discussion

In this study we describe a behavior of euglossine bees dubbed ‘tongue flicking’, which differs from the nectar dehydration practiced by other bees. Nectar is not exposed at the tip but at the base of the proboscis, involving rhythmic movements of a fluid film stretched out between the proximal parts of the proboscis, which are held spread apart for this purpose. Exposing the regurgitated droplets at the base of the proboscis and the splitting of the suction canal into its parallel parts likely circumvents viscosity constraints that would otherwise impede nectar dehydration in these extremely long-tongued bees. The behavior led to higher sugar concentrations of crop content (reaching values up to over 70%, similar to maximal values reported by Wittmann and Scholz [9] for male *Xylocopa nigrocincta*, 66.1% sugar), with the tongue flicking duration being positively correlated to the resulting sugar concentration. The maximal difference between initial and resulting sugar concentration might be overestimated to a small degree, as bees that had not shown tongue flicking behavior nevertheless had slightly higher crop sugar concentrations than what was offered. This could be due to a mixing of the imbibed sugar-water with the residual, concentrated crop content from the previous evening (the crop lining is almost impermeable [45]). Male bumble bees have been shown to exhibit long flights before nectar uptake in the mornings, which was most probably fueled by such residual sugar reserves [11]. Orchid bee tongue flicking duration was not adjusted to the sugar concentration offered in the feeders, which, together with the fact that all observed individuals showed the behavior on at least two of six days, suggests that the behavior is generally shown after feeding (bees that did not engage in tongue flicking were overall inactive). We are unaware of other long-tongued bees employing similar behaviors.

Another behavior which was practiced exclusively by female euglossine bees was the manipulation of mixed nectar-pollen provisions, dubbed ‘kneading’. Pollen foraging cannot be preceded by hour-long nectar dehydration as pollen sources are often depleted quickly following anthesis in the early morning (see review [46]). Additionally, the bees, owing to the long proboscides, cannot regurgitate dehydrated crop content onto pollen loads already positioned in the brood cell, as some short tongued bees do (see e.g. [15]). Instead, female euglossine bees visit nectar flowers during pollen collection trips, and add regurgitated nectar to the pollen loads while hovering, without having displayed the tongue flicking behavior before. Sugar concentration of orchid bee pollen loads nevertheless reached values which were nearly twice as high as the sugar-water offered to the bees. We propose that the extensive manipulation of pollen loads, ‘kneading’, shown before deposition in the brood cells is the main factor responsible for the rise in sugar concentration. All aspects shown in this behavior, creating a large surface by spreading the pollen loads across the whole ventral surface and four legs, moving and kneading the mass while continuously adding more nectar to it without resulting provisions being rendered extremely fluid, suggest evaporative water loss during the process. This in consequence entails a rise in relative sugar content. Though soluble sugars originating from the pollen surface itself have been documented for some plant species, they usually constitute only a small proportion of pollen weight [47]. Supporting our conclusions, honey bee collected pollen (mixed with regurgitated nectar) has a higher sugar content than hand collected pollen [48], [49], and a study on pollen provisions of *Megachile rotundata* and alfalfa pollen [41] showed that the sugar originating from the pollen itself constituted less than 1% of the total sugar content of the provisions. Dissolved amino acids from the pollen might lead to overestimation of sugar content of the measured pollen provisions since the refractive index is based on all dissolved solids in a solution (see [50]). However, duration of pollen-water-contact was kept short (less than 15 minutes; extraction times for analyses of total amounts of water-soluble amino acids are much longer, see [51]) and if overestimation occurred despite this, it was likely only slight (see [41], [50]).

Being able to raise the sugar concentration and thus energy content of aliments after first ingestion in addition to other mechanisms like nectar throughput seems especially important for suction feeding bees that face the problem of having to ingest nectar of suboptimal/low sugar concentrations. Borrell [24] has argued that suction feeding might have been a consequence of longer proboscides, since anatomical constraints might render suction feeding more effective than lapping in such cases despite the constraint on viscosity, and this derived feeding mode seems to have evolved multiple times for flower-visiting insects with extremely long mouthparts like e.g. butterflies and moths (see Discussion in [24]).

However, the long proboscides enable the euglossine bees to exploit a greater variety of nectar sources, which might be highly advantageous in the Neotropical environment, where stingless bees, through recruitment and sheer numbers, can dominate (and deplete) many abundant resources [52], [53]. Being able to shift to flowers whose nectar is not easily accessible to short-tongued stingless bees could have evolved to evade the competition. Such a partitioning has been suggested to occur even between some meliponine bees. *Melipona fulginosa* has a considerably longer proboscis than *Melipona compressipes*, and was constant to flowers with deeper corolla tubes but significantly lower nectar concentrations (21%) rather than competing with the latter species for flowers in the same locality that offered higher concentrated but easily accessible nectar rewards (60% [3]). It seems to be a common phenomenon that plants with deep floral morphologies tend to provide more dilute nectars. Plowright [54] argued that the dilute nectars might simply be a consequence of the deep corolla tubes, since the secreted nectar is protected from evaporation, and other studies support the effect of microclimatic protection on nectar concentration [55], [56]. Though it seems that long-tongued insects exploiting nectar resources from deep tubes face no disadvantage concerning the suction feeding constraint on viscosity as the nectar provided is relatively dilute, lower sugar concentrations for optimal energy intake rate could be expected to limit the efficiency at or the number of alternative nectar sources offering higher concentrations. Both aspects combined would be disadvantageous for highly active organisms if no possibilities to compensate the lower energy uptake were present.

We suggest that orchid bees, in concert with the long tongues and derived feeding modality that enables them to exploit long tubed flowers, have evolved the derived and, in respect to brood provisioning, possibly unique behaviors leading to a higher sugar content of aliments for themselves and their brood, which help to offset the disadvantageous constraints of suction feeding.

## Supporting Information

Video S1 **A male** ***E. dodsoni*** **showing tongue flicking behavior.**
(MP4)

Video S2 **A female** ***E. viridissima*** **adding a droplet of regurgitated crop content to the pollen mass on her corbiculae, slow motion video (factor 12).**
(MP4)

Video S3 **Kneading behavior by a female** ***E. viridissima*** **, slowed down by a factor of 12.**
(MP4)
